# Dietary antarctic krill improves antioxidant capacity, immunity and reduces lipid accumulation, insights from physiological and transcriptomic analysis of *Plectropomus leopardus*

**DOI:** 10.1186/s12864-024-10099-3

**Published:** 2024-02-26

**Authors:** Mengya Wang, Shaoxuan Wu, Hui Ding, Mingyi Wang, Jiayi Ma, Jie Xiao, Bo Wang, Zhenmin Bao, Jingjie Hu

**Affiliations:** 1 MOE Key Laboratory of Marine Genetics and Breeding, College of Marine Life Sciences/Key Laboratory of Tropical Aquatic Germplasm of Hainan Province, Sanya Oceanographic Institution, Ocean University of China, Qingdao, Sanya China; 2 Hainan Yazhou Bay Seed Laboratory, 572025 Sanya, China

**Keywords:** *Plectropomus leopardus*, *Euphausia superba*, Lipid reduction, Liver, Intestine

## Abstract

**Background:**

Due to its enormous biomass, Antarctic krill (*Euphausia superba*) plays a crucial role in the Antarctic Ocean ecosystem. In recent years, Antarctic krill has found extensive application in aquaculture, emerging as a sustainable source of aquafeed with ideal nutritional profiles. However, a comprehensive study focused on the detailed effects of dietary Antarctic krill on aquaculture animals, especially farmed marine fishes, is yet to be demonstrated.

**Results:**

In this study, a comparative experiment was performed using juvenile *P. leopardus*, fed with diets supplemented with Antarctic krill (the krill group) or without Antarctic krill (the control group). Histological observation revealed that dietary Antarctic krill could reduce lipid accumulation in the liver while the intestine exhibited no obvious changes. Enzyme activity measurements demonstrated that dietary Antarctic krill had an inhibitory effect on oxidative stress in both the intestine and the liver. By comparative transcriptome analysis, a total of 1,597 and 1,161 differentially expressed genes (DEGs) were identified in the intestine and liver, respectively. Functional analysis of the DEGs showed multiple enriched terms significantly related to cholesterol metabolism, antioxidants, and immunity. Furthermore, the expression profiles of representative DEGs, such as *dhcr7*, *apoa4*, *sc5d*, and *scarf1*, were validated by qRT-PCR and fluorescence in situ hybridization. Finally, a comparative transcriptome analysis was performed to demonstrate the biased effects of dietary Antarctic krill and astaxanthin on the liver of *P. leopardus*.

**Conclusions:**

Our study demonstrated that dietary Antarctic krill could reduce lipid accumulation in the liver of *P. leopardus*, enhance antioxidant capacities in both the intestine and liver, and exhibit molecular-level improvements in lipid metabolism, immunity, and antioxidants. It will contribute to understanding the protective effects of Antarctic krill in *P. leopardus* and provide insights into aquaculture nutritional strategies.

**Supplementary Information:**

The online version contains supplementary material available at 10.1186/s12864-024-10099-3.

## Introduction

With the world population increasing, the contradiction between land resources and human beings has increased prominently, which caused food shortages and security risks [1, 2]. In this situation, “Blue food”, defined as refers to a range of nutritionally valuable aquatic foods obtained from marine and freshwater sources, encompassing a wide variety of organisms from fish and shellfish to seaweeds, has played an increasingly vital part in human consumption of food [3–6]. Meanwhile, aquaculture has developed rapidly to expand the production of “blue food” to meet increasing population- and wealth-driven demand [7, 8]. In the period 2001–2018, aquaculture has become the fastest-growing sector for “blue food” production with an average annual growth rate of 5.3% and will rise to 50% by 2030 in the contribution to the global “blue food” market [7]. However, an explosive expansion in the size of the aquaculture industry has accelerated the demand for aquafeed supply that relied on fish meal and terrestrial crop ingredients, which will raise concerns about the sustainability of the aquaculture industry [9, 10]. Fish meal production faces challenges in meeting the escalating demand, leading to rising prices [11]. Simultaneously, intensified crop production for aquaculture contributes to excessive waste, amplifying the environmental burden [12]. Furthermore, traditional aquafeed is increasingly unable to meet the demand for healthy growth of aquaculture organisms under the intensive and high-density aquaculture patterns, which usually lead to physiological alterations of aquaculture organisms, such as oxidative stress, metabolism disturbances, and inflammatory responses [13, 14]. Therefore, searching for efficient dietary supplements or alternative ingredients is a priority to ensure the healthy and sustainable development of the aquaculture industry.

Antarctic krill, thriving in the Antarctic Ocean, stands as Earth’s most abundant wild animal, playing a pivotal role in the Antarctic Ocean ecosystem due to its colossal biomass [15, 16]. Boasting high levels of protein, EPA, DHA, vitamins, and phospholipids, Antarctic krill products are gaining popularity in human consumption [17, 18]. Much research has demonstrated that krill products in feeding with a high-fat diet have been demonstrated to improve dyslipidemia, body weight, and glucose metabolism [19, 20]. Within the aquaculture industry, Antarctic krill products have also been recognized as a sustainable source of aquafeed with ideal nutritional profiles [21, 22]. With widespread applications of krill products in the aquaculture industry, great efforts have been made to uncover the detailed benefits of dietary Antarctic krill for aquaculture organisms. Notably, studies have shown that dietary Antarctic krill positively affects the growth performance, muscle quality, lipid metabolism, and immunity system of multiple species, including Atlantic salmon (*Salmon salar*), yellowtail (*Seriola quinqueradiata*), European sea bass (*Dicentrarchus labrax*), and rainbow trout (*Oncorhynchus mykiss*) [23–25]. Furthermore, Antarctic krill, with its rich astaxanthin content known for its antioxidant properties, has demonstrated the ability to alleviate oxidative stress in aquaculture organisms [26, 27].. Indeed, while previous research has offered valuable insights through the exploration of physiological indicators, elucidating the molecular regulation of dietary Antarctic krill on aquaculture biological health is important for gaining a deeper understanding of the mechanisms involved, optimizing aquafeed formulations, monitoring health, promoting sustainability, and exploring biotechnological application.

With the development of Next-generation Sequencing, transcriptome has been widely applied for the resolution of genes and molecular networks that function in the key traits in aquaculture organisms [28, 29]. In exploring effects of feeding or feeding supplements on aquaculture organisms, transcriptome has also been proven to be a valuable tool, offering a detailed gene atlas responsible for physiological changes [30, 31]. However, the applications of the transcriptome in investigating the beneficial effects of dietary Antarctic krill for aquaculture organisms remain limited.

The leopard coral grouper (*Plectropomus leopardus*), is mainly distributed in the tropical and subtropical waters of the Western Pacific Ocean [32]. Owing to its vivid pigmentation, high-protein flesh, and palatable taste, *P. leopardus* has become increasingly popular in human consumption worldwide [33]. Consequently, the aquaculture industry of *P. leopardus* has made significant strides in recent years to meet market demand. Nevertheless, there are also several factors restricting the healthy development of the *P. leopardus* aquaculture industry. For one thing, the intensive and high-density aquaculture mode made *P. leopardus* continuously exposed to stressful conditions, which could be detrimental to physiological status, including metabolism, immunity, and antioxidants [34, 35]. Additionally, traditional feeds without supplements may not offer sufficient nutrition and protection for *P. leopardus* [36]. Therefore, it is essential to explore suitable feed supplements or alternative feeds to ensure healthy and sustainable development of *P. leopardus* aquaculture. Although Antarctic krill has been widely used for aquafeed, its effects on the health of *P. leopardus* have yet to be thoroughly investigated. Here, we found that dietary Antarctic krill was beneficial for the intestine and liver of *P. leopardus* based on histological observation and enzyme activity measurement. Further transcriptome analysis was utilized to investigate the molecular regulation of Antarctic krill on the intestine and liver of *P. leopardus*, and the biased effects of dietary Antarctic krill and astaxanthin on hepatic gene expression of *P. leopardus*. Our findings will facilitate the understanding of the effects of dietary Antarctic krill on *P. leopardus*, and provide valuable insights into the applications of Antarctic krill in *P. leopardus* aquaculture.

## Materials and methods

### Ethics statement

This study was carried out with the permission of the College of Marine Life Sciences, the Ocean University of China Institutional Animal Care and Use Committee on 10 October 2018 (Project Identification Code: 20,181,010).

### Diet preparation, feeding experiment, and sampling

Diet preparation was performed as described previously [37]. Frozen Antarctic krill were bought from Haikou Jianliang Antarctic Shrimp Food Technology Co., Ltd., Hainan, China. Firstly, frozen krill were thawed at room temperature and pulverized thoroughly by the grinder. Subsequently, the basal diet was thoroughly mixed with Antarctic krill and an appropriate amount of tap water until homogenous. Then the mixture was pelleted by a laboratory pellet machine. Meanwhile, the basal diet was also mixed with equal volume water and pelleted using a pellet machine. All diet particles were air-dried and stored at room temperature.

The Antarctic krill feeding experiment was performed at Dongfang Chenhai Aquatic Co., Ltd., Hainan, China. The detailed procedures of the experiment were as follows: A total of 1200 *P. leopardus* larvae with uniform body size (body weight: 0.77 ± 0.29 g) at 60 days after fertilization (dpf) were randomly divided into two groups. For each group, 600 individuals were equally distributed into three tanks (400 L, each) and cultured in recirculating filtered seawater with continuous aeration. Here, two experimental diets were set up: a basal commercial diet (Fish treasure, Dongwan, Japan) without supplements (the control group) and a basal commercial diet supplemented with 50% Antarctic krill (the krill group). Except for diet, the rearing conditions were strictly identical between the control group and the krill group.

After a 40-day feeding trial, the individuals from two groups were anesthetized with MS222. Then the samples of liver and intestine from each individual were collected and washed in RNase-free water. Half of each sample was cut into 5 mm^3^, immediately frozen in liquid nitrogen, and stored at -80 ℃ for further RNA extraction, the remaining was used for histological observation and in situ hybridization. Three individuals were collected in the control group and the krill group, respectively.

### Histological observation

The experimental procedure for histological observation was referred to the protocol of our laboratory [38]. Samples were fixed in 4% paraformaldehyde (PFA) (Boster Biological Technology, USA) overnight, then transferred into methanol (30%, 50%, 70%, 90%, 2 h, respectively), and stored in 100% methanol. After dehydrating in ethanol and embedding in paraffin, the tissue blocks were cut into 5 μm thickness. The haematoxylin-eosin staining experiment was performed according to manufacturer’s specification (Solarbio, Beijing, China). The results were observed and photographed using an Olympus BX43 microscope (Tokyo, Japan).

### Determination of antioxidant enzyme activities of intestines and livers

The analysis of antioxidant enzyme activities was performed as described previously [39]. The frozen intestinal and liver samples were homogenized with 0.9% saline using an electronic homogenizer and centrifugated at 12,000 rpm at 4 ℃, then the supernatant was used for further antioxidant enzyme activities analysis. The enzyme activity of total superoxide dismutase (T-SOD), total glutathione peroxidase (T-GPX), and total antioxidant capacity (T-AOC) levels were measured according to the standard protocol (Beyotime Biotechnology Co., Ltd., Shanghai, China).

### Total RNA extraction, transcriptome library construction, and sequencing

Total RNA extraction of intestines and livers was performed by TRIzol reagent (Invitrogen, Carlsbad, CA, United States) according to the protocols of our lab [40]. After removing genomic DNA by Dnase I (TaKaRa, Dalian, China), the quality and quantity of RNA were evaluated by 1.5% agarose gel electrophoresis and NanoPhotometer Pearl (Implen GmbH, Munich, Germany), respectively. Following re-evaluation using the Agilent 2100 Bioanalyzer (Agilent Technologies, Santa Clara, CA, United States), RNA samples with RIN > 7 were utilized for library construction by Illumina TruSeq RNA Sample Prep Kit (Illumina, San Diego, CA, United States) according to manufacturer’s instruction [41]. Finally, the cDNA libraries were sequenced on Illumina NovaSeq 6000 platform with PE150.

### Transcriptome data processing and analysis

Firstly, the raw reads qualities were checked by fastQC (http://www.bioinformatics.babraham.ac.uk/projects/fastqc/). After trimming adaptors and the low-quality reads by Trimmomatic v0.36, clean reads were obtained. Subsequently, clean reads were mapped to the *P. leopardus* reference genome (NCBI Accession number: PRJNA545594) using HISAT2 v2.1.0 software. The read counts and TPM (Transcripts Per Million) of all genes were calculated by Salmon tools. DEGs analysis was conducted by DEseq2 based on read counts, and genes with|log_2_FoldChange| ≥ 1 and *p*-value < 0.05 were considered as DEGs. The functional analysis of DEGs was performed by DAVID (https://david.ncifcrf.gov/), and results were displayed by a bubble diagram and histogram drawn by R software. Besides, PCA analysis, heatmap, and Venn plot were all constructed by R software.

### qRT-PCR and statistical analysis

The primers used for qRT-PCR were designed by Integrated DNA Technologies (http://sg.idtdna.com/pages/home) based on sequences of genes under test (Table S1). *P. leopardus b2m* gene was used as an internal reference for standardizing expressions of detectd genes [36]. qRT- PCR experiment was performed with a 20 µL mix solution containing 10 µL SYBR qPCR SuperMix Plus (Novoprotein, Shanghai, China), 0.4 µL of each primer (10 µM), 2 µL cDNA template (10 ng), and 7.2 µL nuclease-free water by using LightCycler 480 (Roche, Forrentrasse, Switzerland). The procedure for qRT-PCR was 95 ℃ for 5 min, 45 cycles (95 ℃ for 15 s), and 60 ℃ for 45 s. The relative expressions of detected genes were calculated by 2^−ΔΔCt^ comparative Ct method. Statistical analysis was performed by SPSS20.0 (IBM, NY, USA) with one-way ANOVA followed by the least significant difference test (LSD).

### Dual-color fluorescence in situ hybridization

Fluorescence in situ hybridization (FISH) probes were synthesized using a Digoxigenin (DIG)-labeled RNA labeling kit and a Biotin-labeled RNA labeling kit, respectively, based on the manufacturer’s protocol (Roche, Berlin, Germany). The primers used for template amplification of probes were listed in Table S2. The procedures of FISH were performed following the standard protocols of our lab. The results were observed and imaged by Olympus FV3000 confocal microscope (Olympus, Japan).

## Results

### Effects of dietary Antarctic krill on intestinal and hepatic histology of *P. leopardus* and liver antioxidant status

**Fig. 1:**
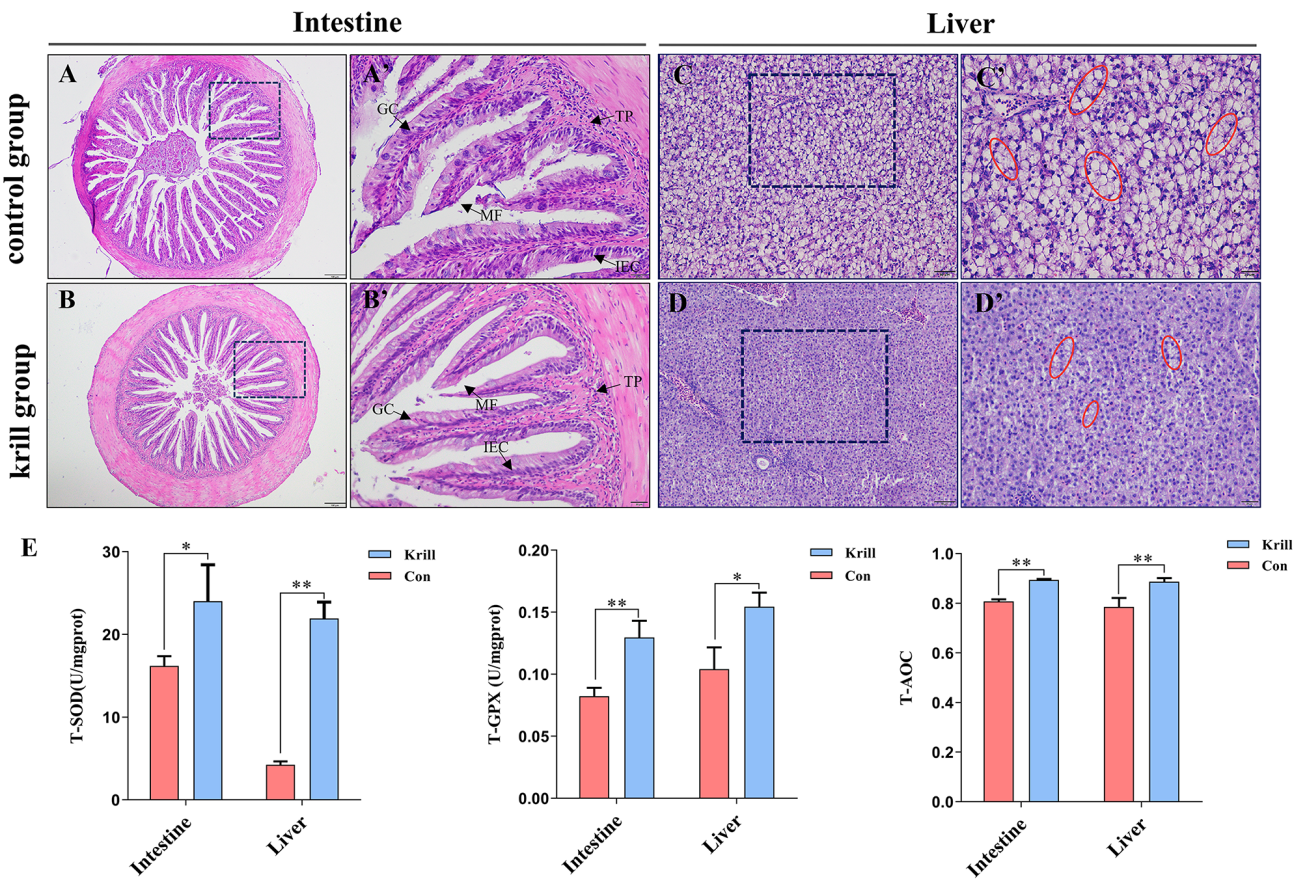
Histological observation of intestine and liver, and antioxidant enzyme activity analysis of the livers with different treatments. (**A-D**) The histology of the intestines and livers with different treatments. (**A and C**) The histology of the intestines and livers in the control group. (**B and D**) The histology of the intestines and livers in the krill group. Scale bars = 50 µm. (**A-D**) The histology of the intestines and livers at higher magnification with different treatments. Scale bars = 20 μm. The vacuolization (lipid droplets) was indicated by circles. MF, mucosal fold; TP, tunica propria; MV, microvillus; IEC, intestine epithelial cells; GC, goblet cell. (**E**) The dynamics of antioxidant capacity of intestine and liver with dietary Antarctic krill. Data were shown as mean ± SEM (*n* = 3). Marks * and ** indicated statistical significance (*P* < 0.05 or *P* < 0.01)

Histological observations of the intestine and liver were carried out and the results were shown in Fig. 1. Notably, no significant morphological differences were observed in the intestines of the krill group compared to the control group (Fig. 1A, B, A and B’), indicating that the effect of feeding krill on the intestinal health of *P. leopardus* did not manifest in histological morphology. However, a noticeable reduction in cytoplasm vacuolization (lipid droplet) was evident in the krill group compared to the control group (Fig. 1C, D and C’, D’). Additionally, the degree of hepatocyte marginalization was significantly decreased in the krill group. Further, we examined the levels of antioxidant system enzymes in the liver and intestine of *P. leopardus*, including T-GPX, T-AOC, and T-SOD. The results, as depicted in Fig. 1E, showcased a substantial increase in enzyme activities of T-SOD, T-GPX, and T-AOC in the krill group compared to the control group.

### Overview of transcriptome data

In this study, a total of 12 libraries were constructed from both the intestine (control_I1, control_I2, control_I3, krill_I1, krill_I2, and krill_I3) and liver (control_L1, control_L2, control_L3, krill_L1, krill_L2, and krill_L3). The detailed information on transcriptome data was listed in Table S3. Following the removal of adaptors and low-quality reads, an average of 19,143,367 clean reads were obtained, with an average Q20 and Q30 of 97.36% and 93.23%, respectively. Subsequently, the clean reads were aligned to the *P. leopardus* reference genome, yielding an average mapping rate of 87.13%. The above results indicated the high quality and sufficiency of transcriptome data for subsequent analysis. The transcriptome raw data has been uploaded to the NCBI sequence Read Archive (SRA) with the accession number PRJNA834932.

### DEGs analysis between the control group and the krill group

**Fig. 2:**
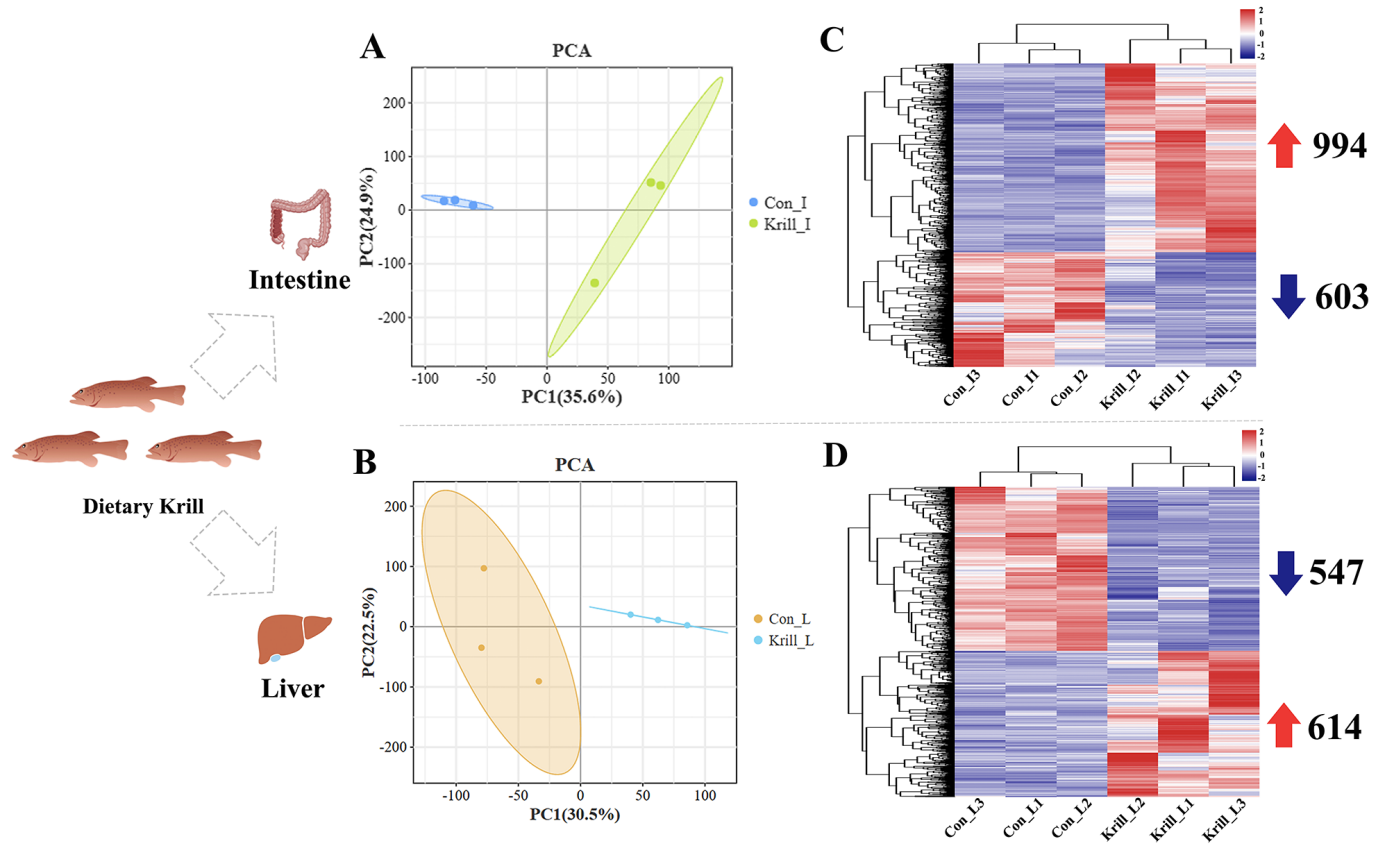
The correlation analysis of transcriptome data and DEGs analysis of intestines and livers with different treatments. (**A**) The PCA analysis of intestines at different treatments. (**B**) The PCA analysis of livers at different treatments. (**C**) Heatmap of all DEGs of intestines between two groups. (**D**) Heatmap of all DEGs of livers between two groups

Firstly, Principal component analysis (PCA) was executed to discern the similarities and differences between samples from the control groups and the krill groups. As depicted in Fig. 2A and B, samples from the same group in both the intestine and liver exhibited distinct clustering. Simultaneously, samples from different groups displayed clear separation in clustering, underscoring the reliability and efficacy of the feeding experiment. Subsequently, two comparison groups were established, and DEGs analysis was conducted by DEseq2. A total of 1,597 and 1,161 DEGs were identified from the intestine and liver, respectively. Besides, the number and expression profiles of up-regulated and down-regulated DEGs in both the intestine and liver were visually represented through by heat-map (Fig. 2C, D).

### Functional analysis of DEGs

**Fig. 3:**
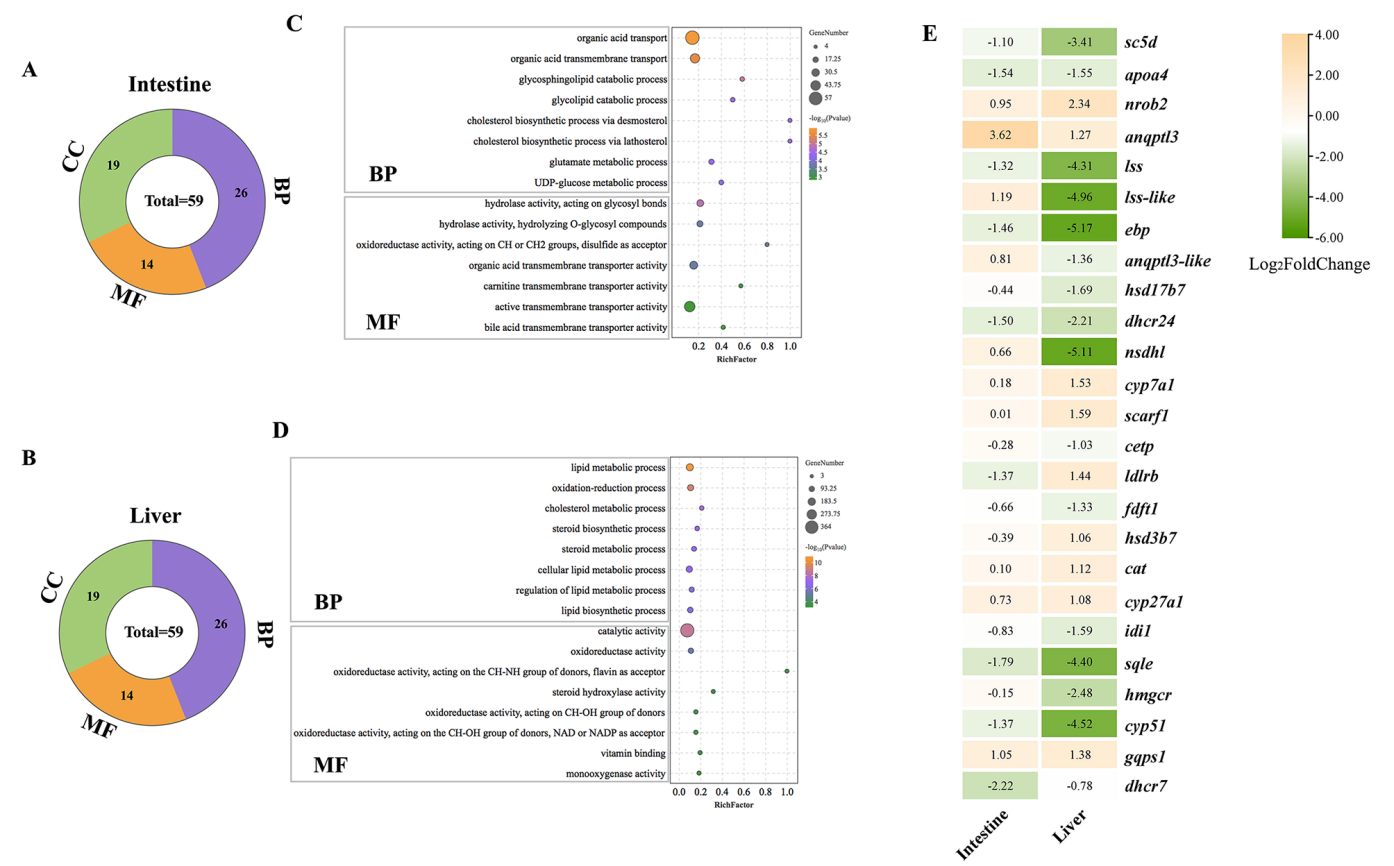
GO enrichment analysis and expression dynamics of DEGs in intestines and livers of *P. leopardus*. (**A**) The number of GO terms enriched at level 2 from different treatments group in intestines. (**B**) The number of GO terms enriched at level 2 from different treatment groups in livers. (**C**) Main biological process terms and molecular function terms of unique DEGs in the control vs. the krill group in intestines of *P. leopardus*. (**D**) Main biological process terms and molecular function terms of unique DEGs in the control vs. the krill group in the livers of *P. leopardus*. (**E**) Heatmap of expression dynamics of cholesterol metabolic related genes in intestine and liver

GO and KEGG enrichment analysis were conducted to unravel the functions of DEGs and the pathways that DEGs were involved in. DEGs obtained from the intestine and liver yield a total of 59 GO terms in level 2, respectively (Fig. 3A, B). Notably, a similarity was observed between categories in biological process (BP), molecular function (MF), and cellular component (CC) enriched from the two group DEGs. At the BP level, the most enriched GO terms were “cellular process”, “single-organism process”, “biological regulation”, and “metabolic process”. At the MF level, DEGs were predominantly associated with “binding”, “catalytic activity”, and “molecular function regulator”. At the CC level, DEGs involved in “cell”, “cell part”, and “organelle” exhibited the highest representation. It was noteworthy that both the DEGs in the intestine and liver were involved in antioxidant activity at the MF level, consistent with the results obtained from the enzyme activity measurement above.

For a deeper understanding of DEGs functions, the terms belonging to BP and MF were further screened. As shown in Fig. 3C, DEGs identified in the intestine were found to be related to the glycolipid catabolic process, cholesterol biosynthesis process, and glucose metabolic process at the BP level, suggesting significant regulation of these biological processes in response to dietary Antarctic krill. Furthermore, multiple MF terms, such as hydrolase activity on glycosyl bonds and transmembrane transporter activity, were screened out, indicating that dietary Antarctic krill might alter the metabolism of glucose and cholesterol by regulating the activity of hydrolase enzymes and transmembrane transport proteins. In the liver, DEGs were found to be extensively involved in biological processes related to lipid metabolism, as well as oxidation-reduction (Fig. 3D). At the MF level, DEGs were notably related to catabolic activity, oxidoreductase activity, steroid hydrolase activity, and vitamin binding. Subsequently, the expression dynamics of cholesterol metabolic-related genes with dietary Antarctic krill were analyzed, revealing that cholesterol synthesis-related genes, including *sc5d*, *dhcr24*, *ebp*, *dhcr7*, and *apoa4*, were almost all down-regulated in both intestine and liver with dietary Antarctic krill (Fig. 3E).

**Fig. 4:**
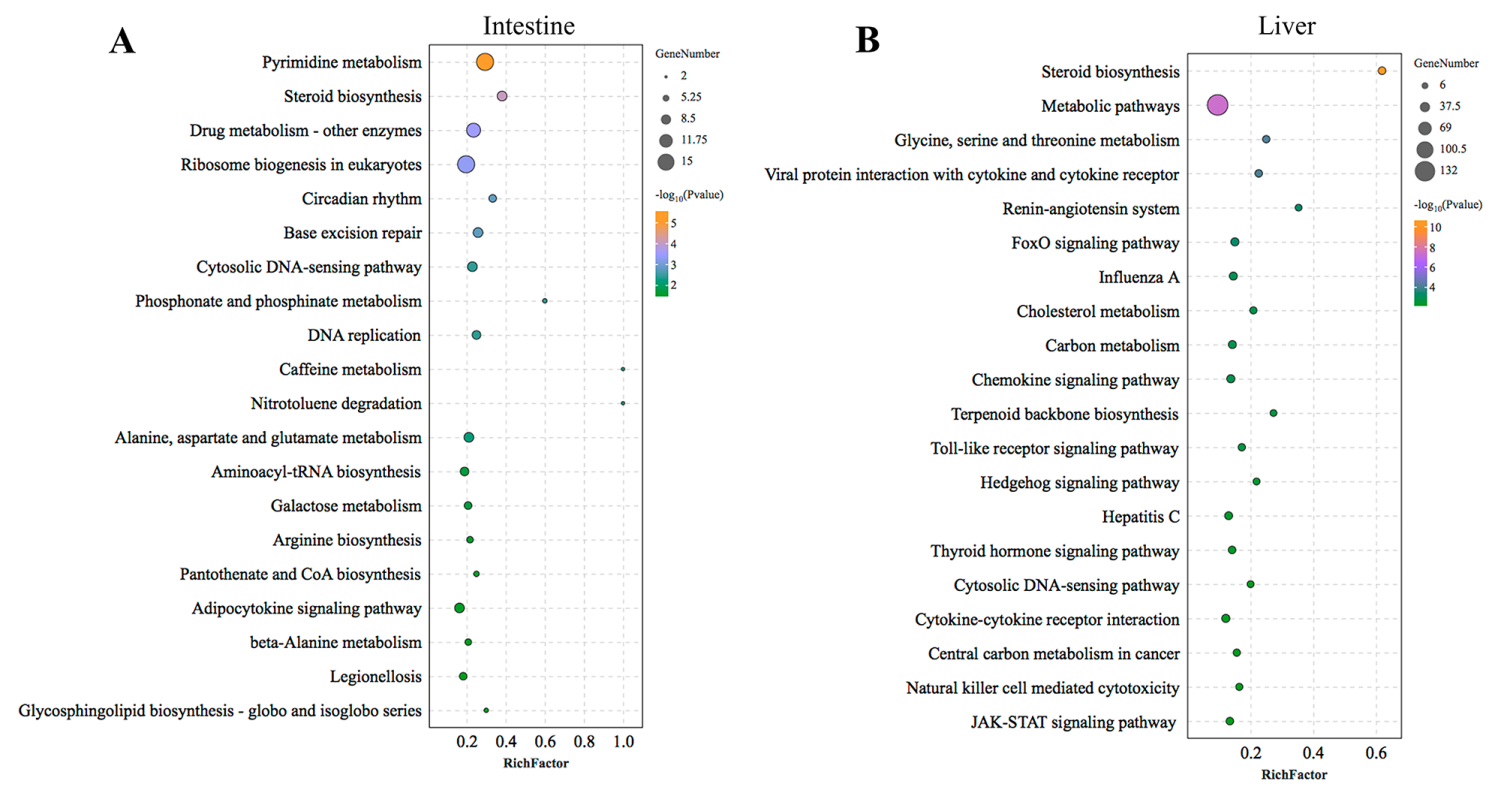
Scatter plots of enriched KEGG pathways for DEGs with different treatments in intestines and livers. (**A**) Scatter plots of enriched KEGG pathways for DEGs in intestines. (**B**) Scatter plots of enriched KEGG pathways for DEGs in livers. Rich Factor was the ratio of the number of DEGs for certain KEGG over the total of genes in that pathway. The significance of identified KEGG pathways was determined by *q*-value < 0.05

The KEGG enrichment analysis results indicated that DEGs from both the intestine and liver were broadly involved in metabolism-related pathways, such as steroid biosynthesis, galactose metabolism, amino acid metabolism, and cholesterol metabolism, which demonstrated that dietary Antarctic krill significantly altered the metabolism state of *P. leopardus*. Moreover, it was found that DEGs from the liver were closely related to immune response-related pathways, including chemokine signaling pathway, Toll-like receptor signaling pathway, Natural killer cell-mediated cytotoxicity, and JAK-STAT signaling pathway (Fig. 4). In the pathways related to immune system, DEGs inhibiting inflammation, such as *pik3rl* and *il17ra*, were up-regulated in the krill group, while some pro-inflammatory factors like *tlr5* and *cxcl10*, were down-regulated in the krill group.

### Validation of DEGs expression profile by the qPCR and FISH

**Fig. 5:**
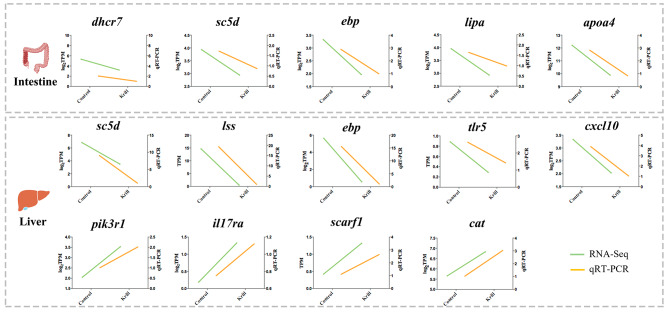
Validation of RNA-seq data by qPCR of representative DEGs intestines and livers. qPCR data were shown as means ± SEM (*n* = 3) from the same three biological replicates utilized in RNA-seq

The results demonstrated that the expression patterns of DEGs obtained from qRT-PCR analysis were generally consistent with those from RNA-seq data (Fig. 5), indicating the trustworthiness of transcriptome data and analysis in our study. In the intestine, cholesterol metabolism-related genes, including *dhcr7*, *sc5d*, *ebp*, *lipa*, and *apoa4*, exhibited a down-regulated in the krill group compared to the control group. In the liver, genes related to cholesterol metabolism, such as *dhcr7*, *sc5d*, and *lss*, also showed a relatively lower expression level in the krill group in contrast to the control group, while *scarf1* and *cat* were up-regulated with dietary Antarctic krill. For immune response-related genes, there was a down-regulation observed in *tlr5* and *cxcl10*, coupled with an up-regulation in *pik3r1* and *il17ra* in the liver under the influence of dietary Antarctic krill.

**Fig. 6:**
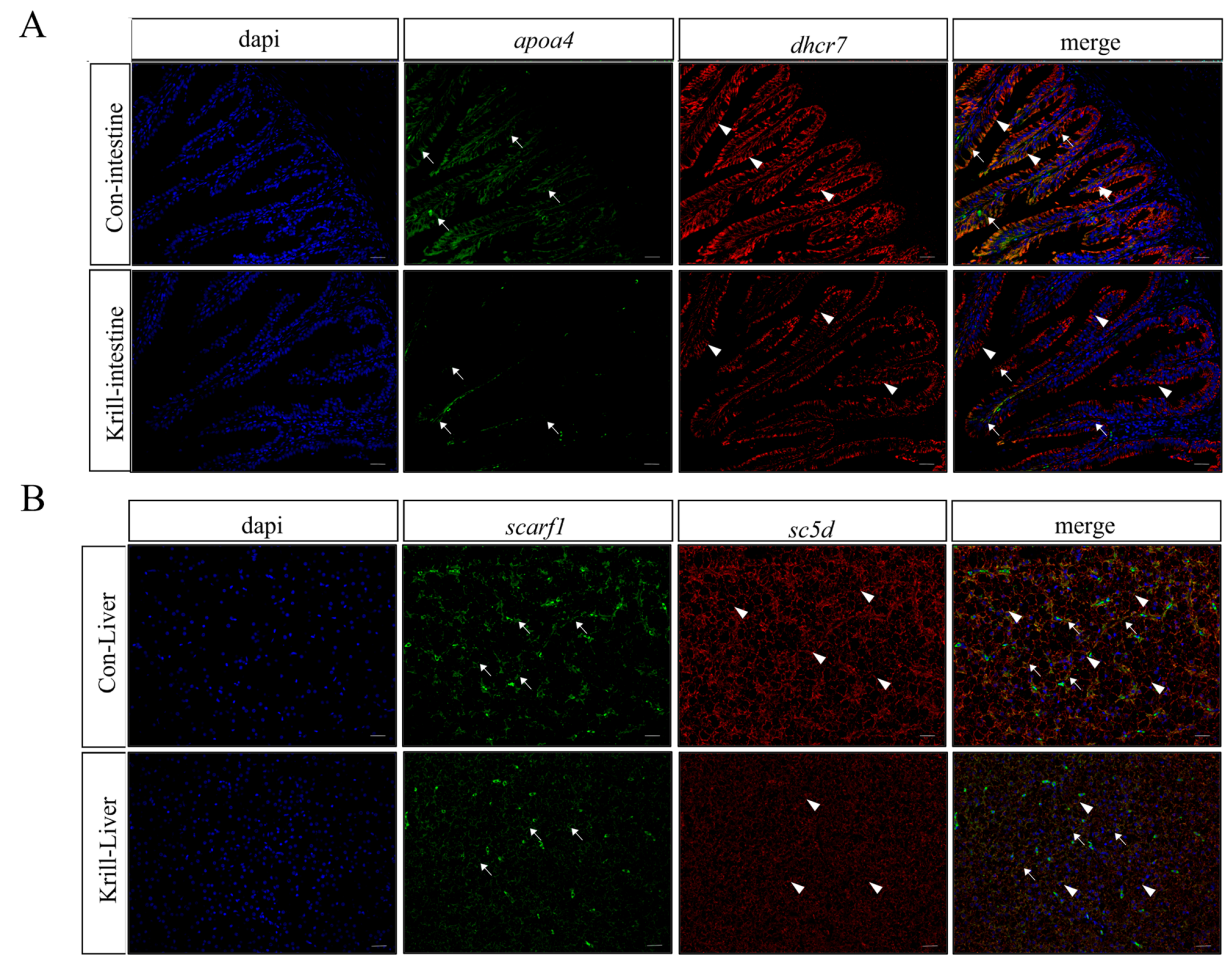
The expression profiles of representative DEGs of intestines and livers. (**A**) Double color fluorescence in situ hybridization verifies the location of *apoa4* and *dhcr7* expression in the intestines of *P. leopardus*. (**B**) Double color fluorescence in situ hybridization verifies the expression and location of *scarf1* and *sc5d* in the livers of *P. leopardus*. The DIG-labeled positive signals were marked by white arrows, and the Biotin-labeled positive signals were marked by white arrowheads. Scale bar: 20 μm

We conducted further validation to explore the expression patterns of DEGs in the intestines and livers of *P. leopardus* using FISH. The nuclei of intestinal epithelial cells (IECs) and hepatocytes were vividly visualized by staining with DAPI. Our findings revealed that the mRNA signals of *apoa4* and *dhcr7* were noticeably diminished in the krill group compared to the control group, as illustrated in Fig. 6A. Intriguingly, these two genes exhibited consistent expression locations in the intestine of *P. leopardus*, with the mRNA signals of *apoa4* and *dhcr7* predominantly localized in the cytoplasm of IECs. Likewise, the expression level of *scarf1* and *sc5d* was stronger in the liver of the control group, and the mRNA signals of *scarf1* and *sc5d* were co-located in the cytoplasm of hepatocytes of *P. leopardus* (Fig. 6B).

### Biased effects of dietary Antarctic krill and astaxanthin on hepatic gene expression of *P. Leopardus*

**Fig. 7:**
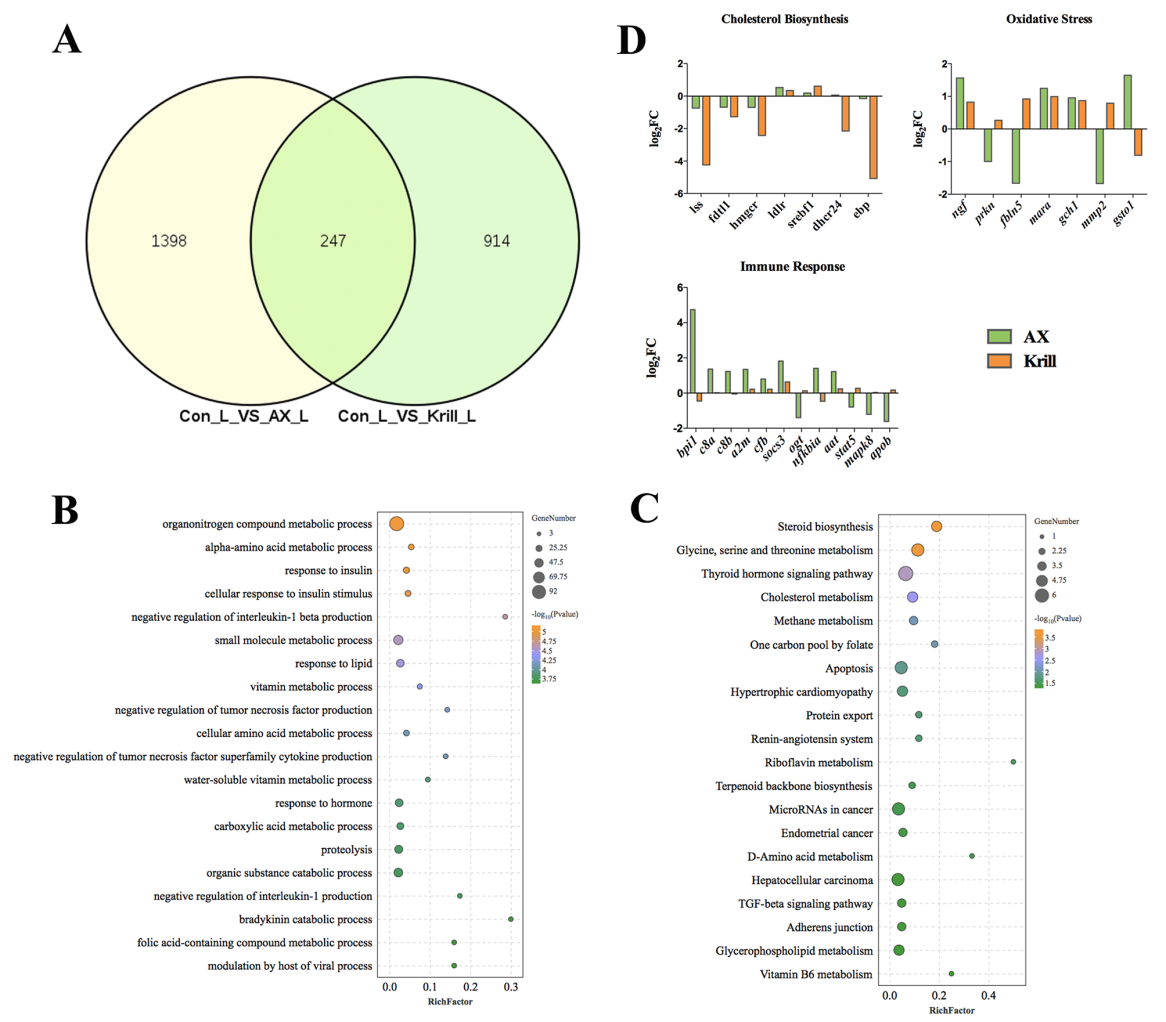
Comparison of effects of dietary Antarctic krill and astaxanthin on gene expression profiles of *P. leopardus* liver. (**A**) Venn diagram of the distribution of the DEGs of *P. leopardus* liver on dietary Antarctic kill and astaxanthin. (**B**) Scatter plots of enriched GO enrichment pathways of DEGs from two groups. (**C**) Scatter plots of enriched KEGG pathways for DEGs from two groups. The Rich Factor was the ratio of the number of DEGs for certain GO and KEGG over the total of genes in that pathway. The significance of identified GO and KEGG pathways was determined by *p*-value < 0.05. (**D**) Gene expression in main biological process terms of unique DEGs in the krill group VS the astaxanthin

Krill stands out as a primary source of astaxanthin, a compound widely recognized in aquaculture for its beneficial properties. In our recent work, we unveiled the positive impact of dietary astaxanthin on the liver health of *P. leopardus*, specifically in its role in regulating lipid metabolism, fortifying the antioxidant system, and enhancing the immune system [36]. Building upon these findings, we delved into a comparative exploration of the effects exerted by dietary Antarctic krill and astaxanthin on the gene expression profiles within the liver of *P. leopardus*. Our results spotlighted 247 genes that exhibited significant differences in expression levels in response to both dietary Antarctic krill (the krill group) and astaxanthin (the AX group) (Fig. 7A). The GO analysis illuminated the substantial involvement of these genes in biological processes linked to lipid metabolism, antioxidant system, and immune responses (Fig. 7B). Furthermore, KEGG enrichment analysis also yielded a bunch of lipid metabolism-related signaling pathways, such as steroid biosynthesis, cholesterol metabolism, and vitamin B6 metabolism, et al. (Fig. 7C). Subsequently, we drew comparisons in the dynamic expression patterns of genes associated with cholesterol metabolism, immune response, and oxidative stress between the krill or AX group, and the control group (Fig. 7D). Compared to the AX group, dietary Antarctic krill showed a more distinct inhibitory effect on cholesterol biosynthesis genes in the liver. Conversely, oxidative stress and immune response-related genes showcased a heightened sensitivity to dietary astaxanthin compared to dietary Antarctic krill, indicating a biased effect of dietary Antarctic krill and astaxanthin on the hepatic gene expression of *P. leopardus*.

## Discussion

The intestine and liver, vital organs responsive to diets, play crucial roles in metabolism, synthetics, and immunity [42, 43]. Based on histological observation, we found that the structural integrity and composition of the intestine remained unaffected by dietary Antarctic krill, aligning with findings in large yellow croaker where Antarctic krill had no discernible impact on intestinal morphology [44]. The intestine has been shown owing high plasticity, defined as the ability to modify its size or thickness and intestinal cells to adapt to different nutritional conditions [45]. Additionally, tightly arranged intestine epithelial cells could be able to maintain the stability of intestinal morphology and structure [46]. In contrast, the integrity of fish liver has been demonstrated to be sensitive to the consumption of formulated feed [47]. Our results showed that a reduction in hepatic cytoplasmic vacuolization in the krill group, suggesting that dietary Antarctic krill could significantly reduce lipid accumulation in the liver. The nutritional composition of Antarctic krill, particularly its omega-3-rich phospholipids, has been recognized for its cholesterol-lowering effects [48]. Indeed, dietary Antarctic krill has also been demonstrated to reduce liver cholesterol and increase free fatty acid contents in multiple aquaculture organisms, including European sea bass, Atlantic salmon, and White shrimp [24, 49, 50]. Furthermore, the results of antioxidant enzyme activity analysis showed that dietary Antarctic krill could significantly reduce oxidative stress in both intestine and liver of *P. leopardus*, which could be attributed to the high contents of astaxanthin that were notable as an efficient antioxidant in Antarctic krill.

The reduction in liver lipid accumulation, modulation of antioxidant capacities, and molecular-level improvements in lipid metabolism and immunity underscored the potential of Antarctic krill as a beneficial dietary supplement. Subsequently, a comparative transcriptome was performed and a bunch of DEGs was obtained. As shown in Fig. 2, a higher abundance of DEGs was identified in the intestine compared to the liver, indicating that dietary Antarctic krill did not elicit significant histological changes in the intestine, it exerted a substantial molecular impact at the transcriptomic level. The functional analysis of these DEGs revealed a pronounced association with lipid metabolism, encompassing cholesterol metabolism, steroid metabolism, and steroid biosynthesis. For instance, 7-dehydrocholesterol reductase (*dhcr7*), known as a terminal enzyme of cholesterol synthesis [51], was found to be down-regulated in the intestine of *P. leopardus* with dietary Antarctic krill, indicating the inhibition effects of Antarctic krill on cholesterol biosynthesis in the intestine. Besides, sterol C5-desaturase (*sc5d*) was also significantly down-regulated in the liver with dietary Antarctic krill, which has been demonstrated to play vital roles in cholesterol biosynthesis [52]. In comparing the intestine and liver, DEGs enriched in the liver were associated with multiple pathways related to immunity, including the Toll-like receptor pathway, Chemokine signaling pathway, Natural killer cell-mediated cytotoxicity, and JAK-STAT signaling pathway, suggesting that the liver was more sensitive to dietary Antarctic krill compared to the intestine in terms of the immune response. Indeed, the protective effects of dietary Antarctic krill on the immune system have been widely demonstrated in multiple aquaculture organisms, such as White shrimp, Japanese flounder, and Red swamp crayfish [37, 53, 54]. In addition, various anti-inflammatory factors have been screened out, the expression dynamics of which have been validated by qRT-PCR. For example, scavenger receptor class F member 1 (*scarf1*), identified for its immune-regulatory role in hepatocellular carcinoma and promote the adhesion of CD4 + T cells to hepatic sinusoidal endothelium in mammals [55]. In teleost, *scarf1* has also been known as an important immunity-related gene in multiple species, such as Common carp, Japanese flounder, and Zebrafish [56–58]. In our study, *scarf1* was found to be distributed in the hepatocytes and up-regulated with dietary Antarctic krill, suggesting that it could function in hepatic immune response in *P. leopardus.* Moreover, other immunity-related genes, including *tlr5*, *pik3r1*, *il17ra*, and *cxcl10*, vital for teleost immune response [59–62], exhibited dynamic regulation in the liver with dietary Antarctic krill. The elucidation of these pathways and gene sets provides a foundation for the development of targeted interventions. Of course, while shedding light on these molecular mechanisms, the detailed functions of these genes and pathways in the intestine and liver warrant further investigation in future research.

Among the nutritional profiles of Antarctic krill, astaxanthin has been famous for its antioxidant, anti-inflammation, and lipid-reduction, which has been widely applied in aquafeed supplements for various aquaculture organisms, including *P. leopardus* [63]. In our previous study, dietary astaxanthin was found to be beneficial for lipid reduction, immune system, and antioxidant in the *P. leopardus* liver [36]. Here, the identification of genes differentially expressed in both the Antarctic krill (krill group) and astaxanthin (AX group) groups, specifically related to cholesterol metabolism and immunity, further underscores the multifaceted functions of dietary Antarctic krill and astaxanthin. To provide valuable insights into the selection of focused feeds in *P. leopardus* aquaculture, the expressions of specific genes related to cholesterol biosynthesis, oxidative stress, and immune response were concluded to evaluate the biased effects of dietary Antarctic krill and astaxanthin on the liver. Based on nutritional profiles, except for astaxanthin, Antarctic krill has been also known for its abundance in DHA and EPA, both of which have been previously established for their beneficial role in cholesterol reduction [64]. Our findings indicated that dietary Antarctic krill exhibited more significant inhibitory effects on most DEGs involved in cholesterol biosynthesis compared to dietary astaxanthin. This suggested that the lipid reduction effect of dietary Antarctic krill was more pronounced than that of dietary astaxanthin, potentially attributed to the synergistic actions of multiple nutrients in Antarctic krill. Future research could delve into identifying the most efficient lipid-reducing nutrient within Antarctic krill. Moreover, dietary astaxanthin demonstrated more conspicuous effects on the expressions of DEGs related to oxidative and immune responses. It’s worth noting that organic pollutants, including fluorine and arsenic, have been detected in krill [65, 66], and microorganisms in krill tissues could potentially affect the immune system and antioxidants [67]. Up to date, the development of krill products, such as krill oil and krill meal, could alleviate the effects of these toxicological factors to some extent. However, the balance between processing costs and the nutritional value of krill products remains to be resolved. In conclusion, our study indicated that the differential effects of dietary Antarctic krill and astaxanthin on gene expression in the liver offer a nuanced understanding of their impact on cholesterol metabolism, oxidative stress, and immune responses.

## Conclusion

In summary, the present study has significantly advanced our understanding of the impact of dietary Antarctic krill on the physiological and molecular aspects of juvenile *P. leopardus*. The addition of krill into the diet demonstrated notable benefits, notably reducing lipid accumulation in the liver and enhancing antioxidant capacities in both the intestine and the liver. Importantly, our findings extended beyond mere physiological observations, revealing profound molecular-level improvements in lipid metabolism, immune response, and antioxidant mechanisms. One of the key revelations from our study is the effectiveness of dietary Antarctic krill in modulating specific genes associated with cholesterol biosynthesis and immunity, exemplified by the differential expression of *dhcr7*, *apoa4*, *sc5d*, and *scarf1*. Furthermore, our comparative transcriptome analysis shed light on the biased effects of dietary Antarctic krill and astaxanthin on gene expressions in the liver. Notably, Antarctic krill exhibited a more pronounced inhibitory impact on genes linked to cholesterol biosynthesis, whereas astaxanthin exerted a greater influence on genes associated with immunity and antioxidant responses. This distinction underscored the specificity and versatility of Antarctic krill as a dietary supplement in aquafeed formulations. By elucidating the intricate molecular mechanisms underlying the positive effects of dietary Antarctic krill, our results will offer a foundation for optimizing aquaculture practices and formulating feed regimes that capitalize on the unique benefits provided by Antarctic krill.

### Electronic supplementary material

Below is the link to the electronic supplementary material.

Supplementary Material 1

Supplementary Material 2

Supplementary Material 3

## Data Availability

The Sequence Read Archive (SRA) has been deposited at GenBank in NCBI, and it will be released upon this study publication. The transcriptome data has been available in the NCBI GenBank repository with the accession number PRJNA1021329, please see the link below for details (https://www.ncbi.nlm.nih.gov/bioproject/PRJNA1021329).
