# Supplementary material for: Dietary antarctic krill improves antioxidant capacity, immunity and reduces lipid accumulation, insights from physiological and transcriptomic analysis of Plectropomus leopardus
Source: BMC Genomics. 2024 Feb 26;25:210. doi: 10.1186/s12864-024-10099-3 (PMC10895837; doi:10.1186/s12864-024-10099-3)
Supplement: Supplementary file 1 — Supplementary Material 1 [file 12864_2024_10099_MOESM1_ESM.docx]

| Primers | Sequences | TM |
| --- | --- | --- |
| Ple-dhcr7-qpcr-Fw | GAGTCCATGACTGTTCTACAC | 60℃ |
| Ple-dhcr7-qpcr-Rv | GGCTACCTGAGTGCTAATTG | 60℃ |
| Ple-apoa4-qpcr-Fw | CACAGCTGCTCCATCATTTA | 60℃ |
| Ple-apoa4-qpcr-Rv | TGACAAGTGCAGAGGTTTG | 60℃ |
| Ple-lss-qpcr-Fw | CGATCTGTGGAAAGTGTTATCT | 60℃ |
| Ple-lss-qpcr-Rv | GGATGTGAAGTCCTGGAATG | 60℃ |
| Ple-scarf-qpcr-Fw | CATCAGTGGAGCCTTCTTTAT | 60℃ |
| Ple-scarf-qpcr-Rv | AATCTGGACATCTGCAACC | 60℃ |
| Ple-cat-qpcr-Fw | GCTTGTGGCTTTGCTTATG | 60℃ |
| ple-cat-qpcr-Rv | TCTTGCTCTTCCCGAATTG | 60℃ |
| Ple-sc5d-qpcr-Fw | TGCAACACCCACTTTCAG | 60℃ |
| Ple-sc5d-qpcr-Rv | ACTGGCTTGATTGGTGTATC | 60℃ |
| Ple-cxcl10-qpcr-Fw | TTGGAGCACAAAGAGACTTC | 60℃ |
| Ple-cxcl10-qpcr-Rv | CATGCCAGAGGCAAAGATAA | 60℃ |
| Ple-tlr5-qpcr-Fw | CAACTCAAAGAGAGAACTAGGG | 60℃ |
| Ple-tlr5-qpcr-Rv | TCCACTACAGAAGAGACTTGA | 60℃ |
| Ple-il17ra1a-qpcr-Fw | CGATAAGGGTTGGAAAGTAGAC | 60℃ |
| Ple-il17ra1a-qpcr-Rv | CAACTATCTCTGGGCCTTATTC | 60℃ |
| Ple-pik3r1-qpcr-Fw | GTGGGAGCACAGAATCAAG | 60℃ |
| Ple-pik3r1-qpcr-Rv | CGGTGAGTACAGTTCCATTT | 60℃ |
| Ple-ebp-qpcr-Fw | GAAGTTACATGCCTCATCTAGG | 60℃ |
| Ple-ebp-qpcr-Rv | GAGGGTCTACAAAGCATCAATA | 60℃ |

**Table S1.** The primers used for qRT-PCR.
