# Supplementary material for: Dietary antarctic krill improves antioxidant capacity, immunity and reduces lipid accumulation, insights from physiological and transcriptomic analysis of Plectropomus leopardus
Source: BMC Genomics. 2024 Feb 26;25:210. doi: 10.1186/s12864-024-10099-3 (PMC10895837; doi:10.1186/s12864-024-10099-3)
Supplement: Supplementary file 2 — Supplementary Material 2 [file 12864_2024_10099_MOESM2_ESM.docx]

| Primers | Sequences | TM |
| --- | --- | --- |
| Ple-apoa4-ISH-Fw | ATTTAGGTGACACTATAGAAGAGGAGTCAGAGAGCCAATC | 56℃ |
| Ple-apoa4-ISH-Rv | TAATACGACTCACTATAGGGAGACAACTCAACAACACACA | 56℃ |
| Ple-lipa-ISH-Fw | ATTTAGGTGACACTATAGAAGAGAAGTTTGTGTGTGTGTG | 56℃ |
| Ple-lipa-ISH-Rv | TAATACGACTCACTATAGGGAGACAATGCTAACGTCCTAAC | 56℃ |
| Ple-scarf-ISH-Fw | ATTTAGGTGACACTATAGAAGAGGAGTGTGTGTTCTGTTG | 56℃ |
| Ple-scarf-ISH-Rv | TAATACGACTCACTATAGGGAGACCACTTGTTATTCTGTAGG | 56℃ |
| Ple-sc5d-ISH-Fw | ATTTAGGTGACACTATAGAAGAGCGAGAGACTTAAGGAGTAT | 56℃ |
| Ple-sc5d-ISH-Rv | TAATACGACTCACTATAGGGAGACCAACGCATTGATACAG | 56℃ |
|  |  |  |

**Table S2.** The primers used for template amplification of probes.
