# Supplementary material for: Dietary antarctic krill improves antioxidant capacity, immunity and reduces lipid accumulation, insights from physiological and transcriptomic analysis of Plectropomus leopardus
Source: BMC Genomics. 2024 Feb 26;25:210. doi: 10.1186/s12864-024-10099-3 (PMC10895837; doi:10.1186/s12864-024-10099-3)
Supplement: Supplementary file 3 — Supplementary Material 3 [file 12864_2024_10099_MOESM3_ESM.docx]

| Sample | Raw Reads | Clean Reads | | Raw Base (G) | Clean Base (G) | Effective Rate (%) | Error Rate (%) | Q20 (%) | Q30 (%) | Mapping Ratio (%) |
| --- | --- | --- | --- | --- | --- | --- | --- | --- | --- | --- |
| Con_I1 | 20,967,928 | | 19,010,604 | 6.29 | 5.7 | 90.67 | 0.03 | 97.59 | 93.78 | 85.23 |
| Con_I2 | 21,695,343 | | 19,514,658 | 6.51 | 5.85 | 89.95 | 0.03 | 97.7 | 93.99 | 85.08 |
| Con_I3 | 19,893,767 | | 17,802,238 | 5.97 | 5.34 | 89.49 | 0.03 | 97.78 | 94.12 | 85.28 |
| Krill_I1 | 16,099,327 | | 14,069,710 | 4.83 | 4.22 | 87.39 | 0.03 | 97.82 | 94.26 | 89.30 |
| Krill_I2 | 18,096,588 | | 17,132,830 | 5.43 | 5.14 | 94.67 | 0.02 | 98.05 | 94.61 | 89.45 |
| Krill_I3 | 22,331,667 | | 21,146,023 | 6.7 | 6.34 | 94.69 | 0.03 | 97.25 | 92.78 | 87.38 |
| Con_L1 | 21,815,312 | | 20,234,707 | 6.54 | 6.07 | 92.75 | 0.03 | 97 | 92.36 | 87.47 |
| Con_L2 | 21,120,974 | | 19,638,686 | 6.34 | 5.89 | 92.98 | 0.03 | 97.1 | 92.72 | 86.97 |
| Con_L3 | 20,942,933 | | 18,786,234 | 6.28 | 5.64 | 89.7 | 0.03 | 97.89 | 94.42 | 87.85 |
| Krill_L1 | 22,079,460 | | 20,787,173 | 6.62 | 6.24 | 94.15 | 0.03 | 96.66 | 91.81 | 87.24 |
| Krill_L2 | 22,556,537 | | 21,088,157 | 6.77 | 6.33 | 93.49 | 0.03 | 96.73 | 91.97 | 87.19 |
| Krill_L3 | 21,890,149 | | 20,509,387 | 6.57 | 6.15 | 93.69 | 0.03 | 96.75 | 91.94 | 87.17 |

**Table S3.** Statistics for RNA-seq data.
